# Glioblastoma, *IDH-*wildtype, with a novel *MEF2D-NTRK1* gene fusion: a case report

**DOI:** 10.3389/fonc.2026.1833072

**Published:** 2026-05-28

**Authors:** Anaya Dewey, Joaquina C. Baranda, Wei Zhang, Mohammad Haeri, Brian Milligan, Tolga Tuncer

**Affiliations:** 1 The University of Kansas Cancer Center, Kansas City, KS, United States; 2 Department of Medical Oncology, University of Kansas Medical Center, Kansas City, KS, United States; 3 Pathology and Laboratory Medicine, University of Kansas Medical Center, Kansas City, KS, United States; 4 Department of Neurosurgery, University of Kansas Medical Center, Kansas City, KS, United States

**Keywords:** bevacizumab, GBM, gene fusion, glioblastoma, *MEF2D-NTRK1*, repotrectinib, temozolomide

## Abstract

A woman, in her mid-50s, presented with headache, confusion, and unstable gait due to a large right parietal mass subsequently diagnosed histologically as glioblastoma, *IDH*-wildtype, CNS WHO Grade 4 with an unmethylated *MGMT* promoter. Next-generation sequencing revealed a rare gene fusion involving myocyte enhancer factor 2D and neurotrophic receptor tyrosine kinase 1 (*MEF2D-NTRK1*), informing our therapeutic approach. The patient was treated with temozolomide concurrently with radiotherapy, followed by tumor treating fields with adjuvant temozolomide. This report examines the distinctive molecular profile of this patient’s glioblastoma, including the remarkable *MEF2D-NTRK1* gene fusion, and the treatment options pursued to mitigate disease progression and optimize quality of life. Here, we will discuss the potential for resistance to tyrosine kinase inhibition and the efficacy of first- and second-generation tyrosine kinase inhibitors in relation to this inhibition. Various tyrosine kinase inhibitors, including entrectinib, larotrectinib, repotrectinib, and selitrectinib, along with bevacizumab, were considered to potentially prolong progression-free survival and overall survival and improve quality of life. We expect to highlight the rarity of this case while discussing the effectiveness of second-generation tyrosine kinase inhibitors in high-grade glioblastomas with rare gene fusions. We also hope to identify the appropriate timeline and treatment sequence for post-standard care, given the lack of official guidelines regarding cases this infrequent.

## Introduction

**Figure 1:**
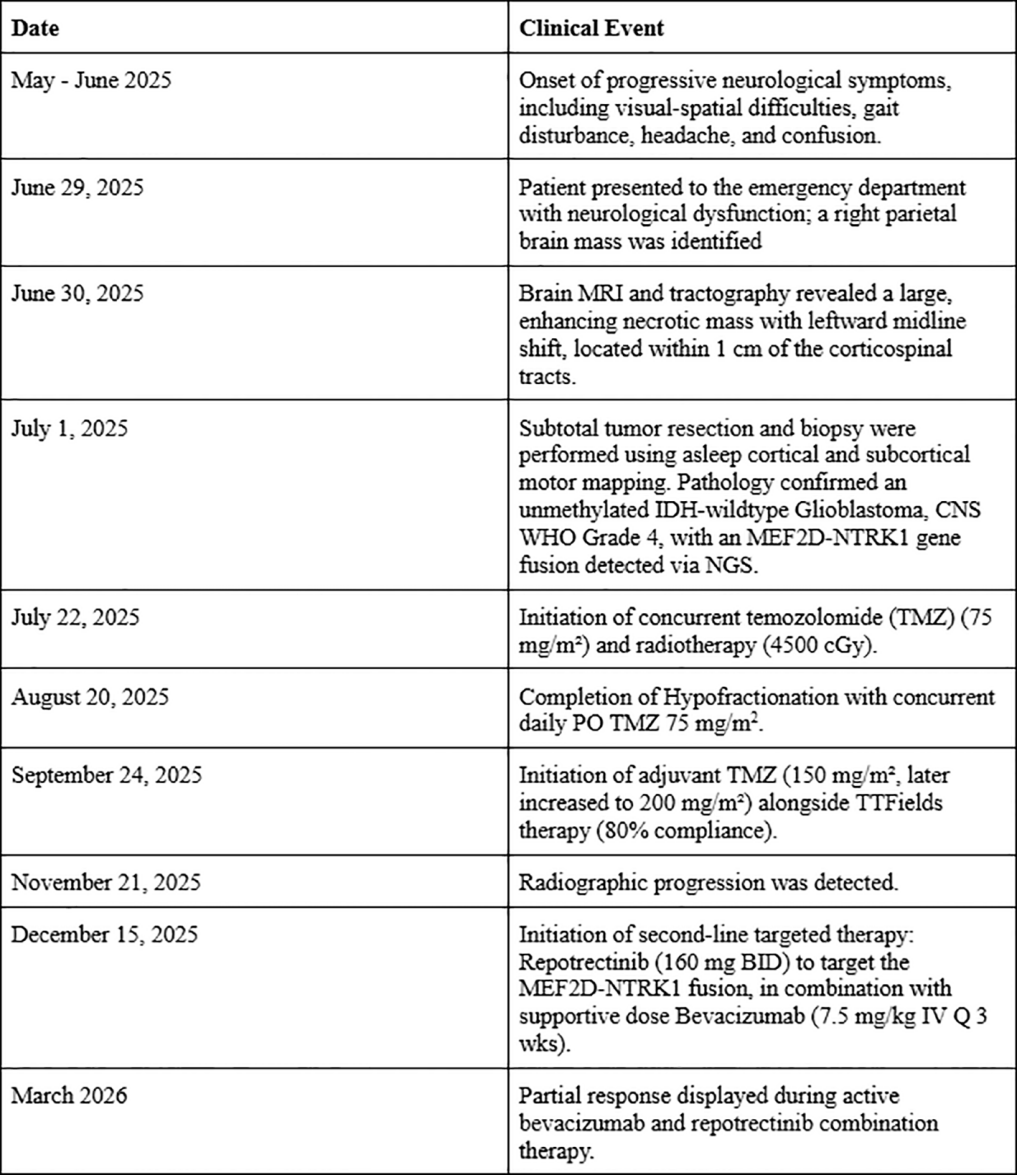
Timeline showcasing treatment management process.

Glioblastoma (GBM) is a highly aggressive, World Health Organization (WHO) grade 4 diffuse glial tumor of the central nervous system. A GBM with NTRK/TRK fusion-positive activity is extremely rare. In a 2018 study examining 11,502 tissue samples for 53 different gene fusions, 32 cases were identified as neurotrophic receptor tyrosine kinase (*NTRK*) gene fusions, with only 0.3% of those cases containing the transcription factor, myocyte enhancer factor 2D (*MEF2D*), and the tyrosine kinase gene, neurotrophic receptor tyrosine kinase 1 (*NTRK1*) (1). Recently, clinicians have utilized the molecular makeup of GBMs to guide individualized treatment decisions (2). Isocitrate dehydrogenase (*IDH*) -wildtype (WT) glioblastomas are molecularly defined by one or more of the following alterations: telomerase reverse transcriptase (*TERT*) promoter mutation, epidermal growth factor receptor (*EGFR*) amplification, or the characteristic whole-chromosome copy-number signature of chromosome 7 gain with chromosome 10 loss (+7/-10) (3). We present a 56-year-old woman with a glioblastoma, *IDH*-wildtype, CNS WHO Grade 4 with unmethylated O (6)-methylguanine-DNA methyltransferase (*MGMT*) promoter exhibiting a unique *MEF2D-NTRK1* fusion. This report aims to evaluate the molecular characterization of this patient’s GBM, including a deeper look at methylation profiling, immunohistochemistry (IHC), and other features, to support more informed clinical decisions (**Figure 1**).

## Case description

A 56-year-old right-handed female with no significant past medical history presented with a four-week history of headaches and a two-week history of gait instability, visual-spatial deficits, and confusion at a local emergency department. Physical examination demonstrated left-sided sensory ataxia and pronator drift.

## Radiologic findings

**Figure 2:**
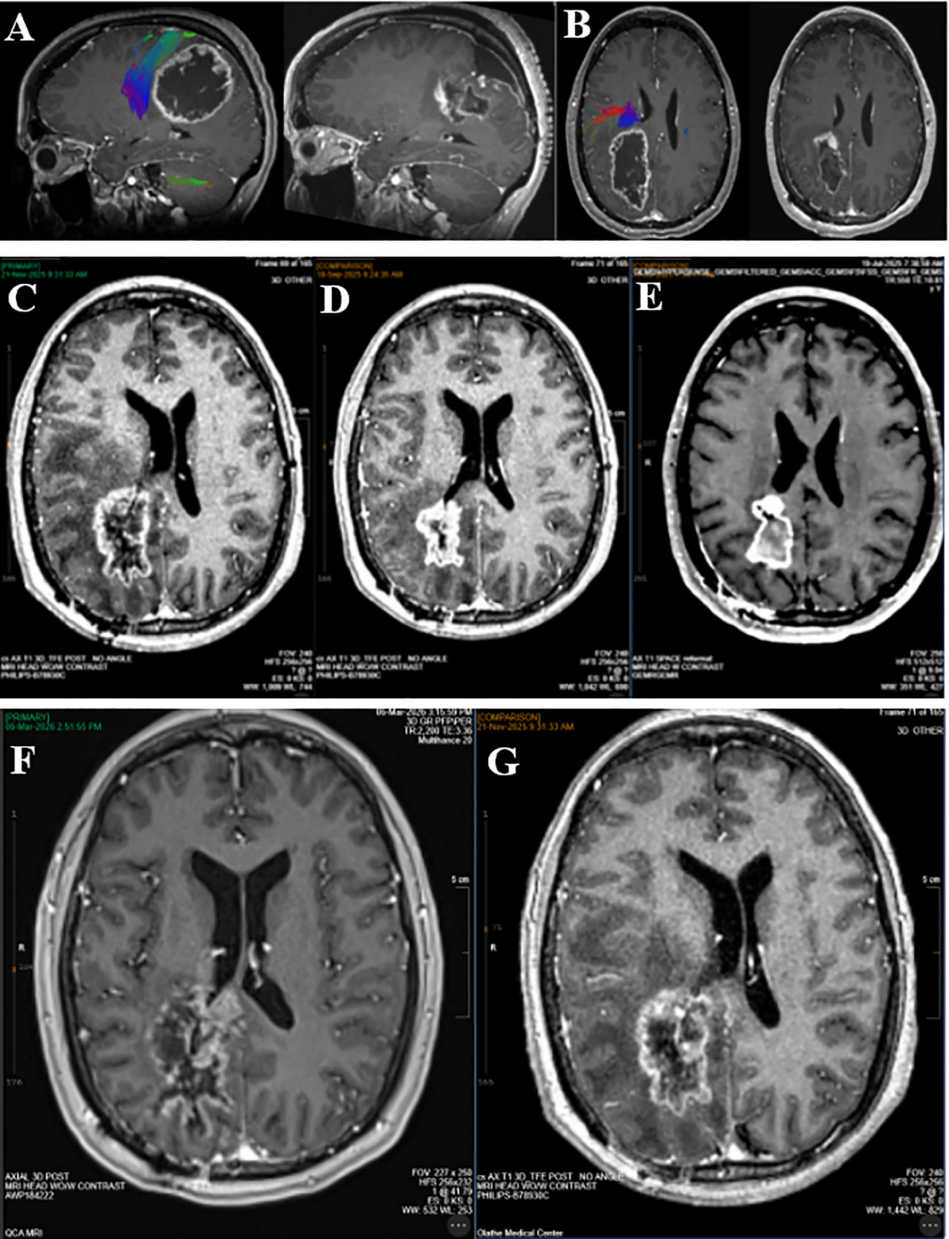
**(A)** Left, preoperative sagittal T1-weighted MRI with contrast. Corticospinal tracts are indicated in blue (superior-inferior oriented) and yellow (anterior-posterior oriented) with intermediate coloration representing oblique trajectory. Right, postoperative sagittal T1-weighted MRI showed the small residual along the anterior-inferior aspect of the cavity. **(B)** Left, preoperative axial T1-weighted MRI with contrast. Corticospinal tracts are indicated in blue (superior-inferior oriented) and red (medial-lateral oriented). Right, postoperative axial T1-weighted MRI with contrast showing the small residual along the anterior margin of the cavity. **(C, D, E)** Various images featuring brain MRI of Glioblastoma patient taken in **(C)** November 2025, displaying progressive disease, **(D)** September 2025, taken post-radiation therapy, and **(E)** July 2025, taken pre-radiation therapy. **(F, G)** Side-by-side comparison of brain MRI taken **(F)** March 2026 and **(G)** November 2025 featuring improvement during Bevacizumab and Repotrectinib combination therapy.

Magnetic resonance imaging (MRI) revealed a 6.7 cm by 3.7 cm ring-enhancing, centrally necrotic right parietal mass suggestive of a high-grade glioma. Diffusion tensor imaging (Tractography) indicated the corticospinal motor tracts were abutting the anterior margin of the lesion (**Figure 2B**).

## Surgical procedure

The patient underwent a right parietal craniotomy for subtotal resection, utilizing intraoperative asleep motor mapping to preserve corticospinal tract integrity. Of note, corticospinal tracts could be occasionally stimulated at 3 mA along the anterior margin, suggesting that primary motor axons were within 3 mm of the resection margin, requiring cessation of resection in that area. After surgery, her neurological deficits nearly resolved (**Figure 2B** - Tractography).

## Pathologic findings

Pathological evaluation confirmed classical features of glioblastoma, including palisading necrosis, microvascular proliferation, and brisk mitotic activity. The integrated diagnosis was glioblastoma, *IDH*-wildtype, CNS WHO Grade 4. Molecular profiling revealed an unmethylated *MGMT* promoter, an intermediate tumor mutational burden (5.71 mut/Mb), a *TERT* promoter mutation (c.-124C>T), and a concurrent gain of chromosome 7 and loss of chromosome 10 (+7/-10). Most crucially, NGS detected an *MEF2D-NTRK1* gene fusion.

## Treatment

Following surgical treatment, a pre-chemoradiation treatment MRI of the brain was captured (**Figure 2E**). The patient, then, completed 3 weeks of concomitant temozolomide (TMZ) (75mg/m2) with 4500 cGy of Intensity-Modulated Photon Radiotherapy (IMRT). A hypofractionated regimen of 4500 cGy in 15 fractions was chosen based on tumor size and logistical considerations as compared to the standard of care (SOC) of 6 weeks with 30 fractions. After completion of successful chemoradiation, adjuvant cyclical PO TMZ was started. Afterward, an MRI of the brain was captured post-radiation therapy (**Figure 2D**). The patient subsequently transitioned to adjuvant cyclic TMZ (initially 150 mg/m2 in cycle 1, increased to 200 mg/m2 in cycle 2) combined with TTFields, achieving an optimal 80% device compliance.

Following three cycles of adjuvant therapy, an MRI in November 2025 demonstrated radiographic progression (**Figure 2C**). It should be noted that it is notoriously difficult to differentiate between pseudoprogression and true progression in the few months after chemoradiation. Given the patient’s increasing steroid dependence, persistent nausea, and imaging findings consistent with progression, a targeted second-line regimen was initiated. The patient was commenced on repotrectinib (160 mg) to specifically target the *MEF2D-NTRK1* fusion, alongside bevacizumab (7.5 mg/kg) to mitigate edema and reduce corticosteroid requirements. The patient has tolerated this combination well for over four months without hematological toxicity. For radiographical assessment, Response Assessment in Neuro-Oncology (RANO) criteria were used (**Figures 2F, G**). Presently, the patient has been on this combination therapy for over four months. Ongoing treatment will continue until progressive disease (PD) occurs. While minimal data are available on the efficacy of combining repotrectinib with bevacizumab, the decision to pursue combination therapy was informed by the presenting symptoms of the patient and the molecular characterization of the glioblastoma, with the aim of symptom control.

## Discussion

This case underscores the clinical utility of comprehensive genomic profiling in *IDH-*wildtype glioblastoma, particularly in identifying rare, albeit actionable, genomic alterations such as the *MEF2D-NTRK1* gene fusion. The tumor exhibited molecular hallmarks of *IDH*-wildtype glioblastoma, including a +7/-10 chromosomal copy number profile, a *TERT* promoter mutation, an unmethylated *MGMT* promoter, and a low tumor mutational burden (TMB) with microsatellite stability (MSS). These features classically portend a poor response to standard alkylating chemotherapy and immune checkpoint blockade (4, 5). NGS revealed an uncommon gene fusion between the transcription factor MEF2D and the receptor kinase *NTRK1*, potentially providing an opportunity for an alternate and rather unique management approach, whether in a *de novo* or salvage setting.

Consistent with the typical clinical course of glioblastoma, the patient experienced disease progression despite receiving standard of care concurrent chemoradiation followed by adjuvant temozolomide and tumor-treating field (TTFields). The standard approach of concurrent TMZ and XRT (radiation therapy) is known as the Stupp protocol (6). Adjuvant TMZ with TTFields was administered following the initial treatment. The EF-14 randomized clinical trial of TTF in addition to maintenance TMZ support improved clinical outcomes in patients treated with TTFields in conjunction with TMZ compared to TMZ alone (7, 8). The identification of an *MEF2D-NTRK1* fusion provided a unique opportunity for targeted salvage therapy. The *NTRK1* gene encodes the TRKA receptor, which regulates essential neuronal survival pathways, including the P13K-Akt and MAPK/ERK signaling cascades. Gene fusions involving *NTRK1*, such as with the transcription factor MEF2D, result in constitutively active kinase domains that drive uncontrolled cellular proliferation and tumor growth.

After tumor progression, TKIs were considered to add a treatment with a different mechanism of action, targeting the MEF2D-NTRK1 gene fusion in the process. TKIs such as entrectinib, larotrectinib, repotrectinib, and selitrectinib were assessed. MEF2 modulates many genes and miRNAs that play a key role in neuronal viability and axonal growth (9). NTRK1 also plays a key role in neuronal survival by encoding the Tropomyosin receptor kinase A (TRKA) receptor, which transduces signals that activate pathways such as PI3K-Akt and MAPK/ERK, thereby preventing neuronal cell apoptosis and promoting neuronal development. When gene fusions occur, the kinase domain within the resulting fusion protein becomes constitutively active, leading to uncontrolled cell growth and proliferation. TKIs inhibit tyrosine kinase activity through various mechanisms. Resistance to TKIs, however, is a common phenomenon through acquired resistance of on- and off-target mutations through modifications to the kinase domain directly or activation of nearby signaling mechanisms (10). We were most interested in a TKI that would effectively inhibit TRKA activity with minimal risk of TKI resistance. First-generation TKIs such as entrectinib and larotrectinib were investigated, with subsequent studies providing evidence of their efficacy in high-grade gliomas. A 2018 study on entrectinib efficacy, comprising clinical findings from various trials including ALKA, STARTRK-1, and STARTRK-2, revealed that the overall response rate for 54 adult patients with NTRK fusion-positive solid tumors, after 15.5 months, was 57.4%. The median investigator-assessed PFS rate was 12 months and 7.7 months, both for subjects diagnosed with a CNS disorder and not. It was concluded that entrectinib was effective through positive clinical outcomes for patients with NTRK-fp solid tumors (11). In a 2024 study highlighting larotrectinib efficacy, compiling data from various clinical trials (NCT02122913, NCT02576431, NCT02637687) and comparing with patients who received non-TRKi therapies, subjects receiving larotrectinib were revealed to have a longer overall survival (OS), PFS, and time to next treatment (TTNT) than those of patients receiving non-TRKi therapies (12). While larotrectinib and entrectinib exhibited promising results for inhibiting TRKA, B, and C kinase activity, patterns of resistance were highly prevalent, with the duration of response (DOR) being limited to a recorded maximum of 30 months (11). First-generation TKIs, such as larotrectinib, inhibit the overactivity of tropomyosin receptor kinases in mutated tumor environments by obstructing the ATP-binding site. Over time, however, tumors gain resistance mechanisms, rendering TKI suppression ineffective. Two resistance mechanisms to note are solvent front and xDFG mutations that cause a conformational change in the ATP-binding site or DFG (Asp-Phe-Gly) motif, preventing larotrectinib binding, and sometimes increasing the overactivity of the tyrosine kinase as well. Second-generation TKIs such as Repotrectinib and LOXO-195 (also known as selitrectinib) can overcome the binding site’s mutational, conformational change by adopting a shape compatible with said site (13–15).

While limited data are available on the effects of second-generation TKIs on an *MEF2D-NTRK1* gene fusion, clinical findings have demonstrated their increased potency against other *NTRK* fusions, suggesting that pursuing them would result in long-term inhibition of this case’s fusion. In a 2024 study involving 12 patients with NTRK-fused gliomas (both high-grade gliomas and low-grade gliomas with NTRK1 or NTRK2 gene fusions present), it was found in one patient with a *ZBTB43-NTRK2* high-grade glioma that received chemotherapy and proton therapy that repotrectinib was most useful in preventing tumor recurrence for multiple years (13). In addition to SOC chemoradiation and TTFields, it is understood that NTRKi could prolong PFS and OS and maximize quality of life (QoL) by controlling tumor growth through a different mechanism. Both first and second-generation TKIs (when addressing gene fusions) are an innovative advancement in precision medicine, as they allow more treatment options for patients with rare conditions such as this case. In the context of a near-uniformly fatal disease like GBM, the integration of targeted therapy is imperative. However, the optimal sequence of targeted treatment remains a critical unknown: it is unclear whether these agents should be deployed immediately as maintenance following standard chemoradiation or reserved as a salvage strategy upon progression or recurrence.

## Patient perspective

The combination treatment was subjectively tolerated well while preserving QoL. Progression has been delayed by at least four months, allowing for improved well-being during treatment.

## Limitations

This report is inherently limited by its single-patient design, which precludes broad generalizability and prevents the definitive attribution of clinical or radiographic improvements solely to repotrectinib, given the concurrent use of bevacizumab. Additionally, the observed clinical gains were modest and partially transient. Finally, in the absence of established neurooncology guidelines for this specific genomic presentation, dosing was extrapolated from other disease processes, leaving the optimal sequencing of this therapeutic strategy as an area requiring further prospective investigation.

## Data Availability

The original contributions presented in the study are included in the article/supplementary material. Further inquiries can be directed to the corresponding author.
